# Supplementary figures and images for: Diclofenac sodium ion exchange resin complex loaded melt cast films for sustained release ocular delivery
Source: Drug Deliv. 2017 Feb 6;24(1):370–9. doi: 10.1080/10717544.2016.1256000 (PMC8253122; doi:10.1080/10717544.2016.1256000)

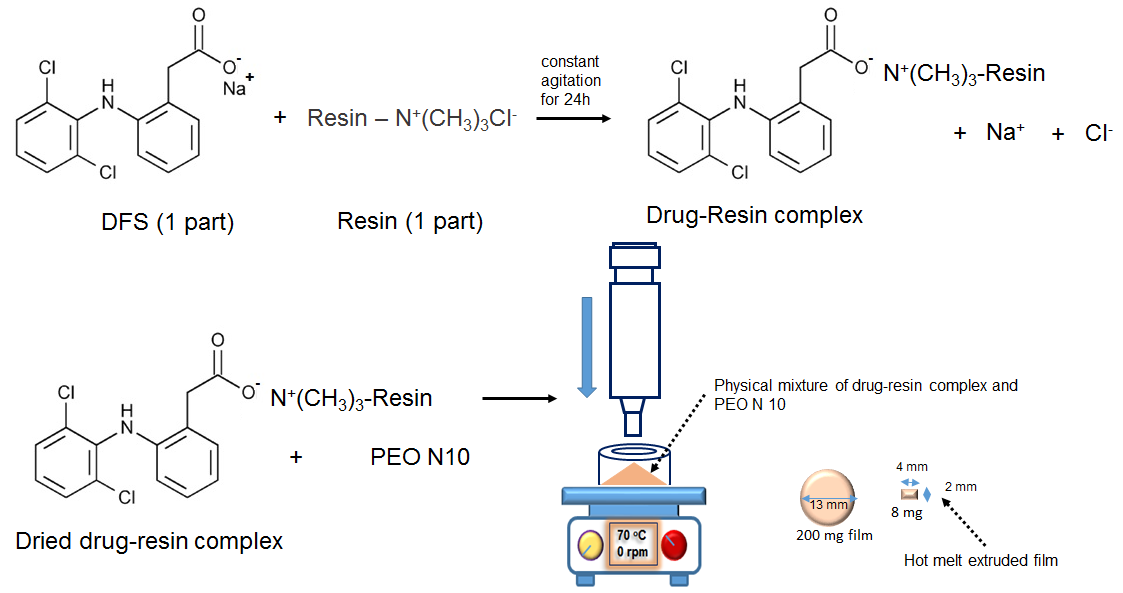

Supplement: Supplimental_data.png [file IDRD_A_1256000_SM2624.png]
